# Combined EEG/MEG Can Outperform Single Modality EEG or MEG Source Reconstruction in Presurgical Epilepsy Diagnosis

**DOI:** 10.1371/journal.pone.0118753

**Published:** 2015-03-11

**Authors:** Ümit Aydin, Johannes Vorwerk, Matthias Dümpelmann, Philipp Küpper, Harald Kugel, Marcel Heers, Jörg Wellmer, Christoph Kellinghaus, Jens Haueisen, Stefan Rampp, Hermann Stefan, Carsten H. Wolters

**Affiliations:** 1 Institute for Biomagnetism and Biosignalanalysis, Westfälische Wilhelms-Universität Münster, Münster, Germany; 2 Institute for Biomedical Engineering and Informatics, Technische Universität Ilmenau, Ilmenau, Germany; 3 Epilepsy Center, Universitätsklinikum Freiburg, Freiburg im Breisgau, Germany; 4 Department of Neurology, Klinikum Osnabrück, Osnabrück, Germany; 5 Department of Clinical Radiology, Universitätsklinikum Münster, Münster, Germany; 6 Ruhr-Epileptology Department of Neurology, Universitätsklinikum Knappschaftskrankenhaus Bochum, Bochum, Germany; 7 Epilepsy Center, Department of Neurology, Universitätsklinikum Erlangen, Erlangen, Germany; Universiteit Gent, BELGIUM

## Abstract

We investigated two important means for improving source reconstruction in presurgical epilepsy diagnosis. The first investigation is about the optimal choice of the number of epileptic spikes in averaging to (1) sufficiently reduce the noise bias for an accurate determination of the center of gravity of the epileptic activity and (2) still get an estimation of the extent of the irritative zone. The second study focuses on the differences in single modality EEG (80-electrodes) or MEG (275-gradiometers) and especially on the benefits of combined EEG/MEG (EMEG) source analysis. Both investigations were validated with simultaneous stereo-EEG (sEEG) (167-contacts) and low-density EEG (ldEEG) (21-electrodes). To account for the different sensitivity profiles of EEG and MEG, we constructed a six-compartment finite element head model with anisotropic white matter conductivity, and calibrated the skull conductivity via somatosensory evoked responses. Our results show that, unlike single modality EEG or MEG, combined EMEG uses the complementary information of both modalities and thereby allows accurate source reconstructions also at early instants in time (epileptic spike onset), i.e., time points with low SNR, which are not yet subject to propagation and thus supposed to be closer to the origin of the epileptic activity. EMEG is furthermore able to reveal the propagation pathway at later time points in agreement with sEEG, while EEG or MEG alone reconstructed only parts of it. Subaveraging provides important and accurate information about both the center of gravity and the extent of the epileptogenic tissue that neither single nor grand-averaged spike localizations can supply.

## Introduction

Noninvasive EEG and MEG are important tools for presurgical epilepsy diagnosis, they can guide the placement of invasive electrodes, the current gold standard in presurgical epilepsy diagnosis, and in some cases they can even supply sufficient information for a surgical intervention without invasive recordings [1–12]. Especially with increasing use of realistic and individual head models, improved MRI co-registration approaches and high sensor numbers, the accuracy and precision of noninvasive source reconstructions have increased notably [13–17]. It has also been recently shown that there is good agreement between noninvasive EEG and MEG source reconstructions and fMRI responses [18].

### Sensitivity differences of EEG and MEG with regard to source location and orientation

EEG and MEG contain complementary information [19]. Although the sources that produce EEG and MEG recordings are the same, their distinct properties cause them to produce different sensor signals. Patients with detectable epileptic activity only in EEG or MEG illustrate the importance of simultaneous measurements for epileptic spike detection [20–22]. Furthermore, with regard to the reconstruction of the sources underlying the measured signals, unlike MEG, which measures almost only quasi-tangential sources, EEG can measure both quasi-tangential and quasi-radial sources [23–30]. When compared to the EEG, MEG is thus also less sensitive to all deeper sources, not only because the signal decays with the square of the distance from the source to the measurement sensors (MEG shares this drawback with the EEG), but also because deeper sources become more quasi-radial. On the other hand, in comparison to EEG, MEG achieves higher SNRs (signal-to-noise-ratios) for more lateral quasi-tangential sources, also because the measured signals are nearly not contaminated by mainly quasi-radial biological noise [31]. The signal topographies of EEG and MEG are almost orthogonal to each other and, because the low skull conductivity smears out the EEG, the distance between the poles of dipolar EEG topographies is in practice greater than for MEG. Therefore, the simultaneous acquisition of EEG and MEG increases the probability of measuring the important aspects of the signal topographies by at least one of the two modalities, thus stabilizing the source reconstructions.

### Sensitivity differences of EEG and MEG with regard to volume conduction

Noninvasive EEG measures the signals passing through the poorly conducting skull, which spatially smoothens and attenuates the electric potentials. On the contrary, the skull conductivity has small to negligible effects on magnetic fields recorded by the MEG. Therefore, EEG is shown to be very sensitive to uncertainty and variations in skull conductivities while MEG is largely insensitive to these changes [29], [32]. Furthermore, studies showing the importance of modeling skull inhomogeneity in EEG address the need for distinguishing the higher resistive skull compacta and lower resistive skull spongiosa compartments [33–35]. Both EEG and MEG are sensitive to differences in the cerebrospinal fluid (CSF), the gray and the white matter compartments, and thus accurate modeling of these compartments is important [29], [36]. White matter is known to be anisotropic and diffusion tensor imaging (DTI) provides the necessary information to model this [37], [38]. Earlier studies revealed that it might be important, especially for deeper sources, to model white matter anisotropy for accurate EEG and MEG source reconstructions [30], [39]. Therefore, in this study, we constructed and used a six-compartment (scalp, skull compacta, skull spongiosa, CSF, gray matter, white matter) finite element (FE) head volume conductor model with anisotropic white matter compartment. In addition, we calibrated the head model by means of adjusting skull conductivity to ensure the best fit to the somatosensory evoked potential and field data to reduce localization errors due to highly unrealistic assumptions on the patient’s individual skull conductivity [40–42].

### Combined EEG and MEG source reconstruction

The different sensitivity profiles and especially the complementarity of EEG and MEG as explained in the previous two paragraphs encourage researchers to increase the synergy by measuring and evaluating EEG and MEG simultaneously [21], [24], [25], [27], [42]. In this paper we investigated if EMEG (combined EEG/MEG) can add additional information to single modality EEG or MEG with regard to source reconstruction of epileptic activity. For this purpose, we especially focused on time instants at spike onset, which on the one hand have limited reliability due to smaller SNRs but which on the other hand are more immune to misleading localizations due to propagation.

### Extent of the epileptogenic tissue

Determining the location and the extent of the epileptogenic tissue is of great importance for successful surgery and seizure freedom. Köhling et al. [43] and Speckmann et al. [44] employed optical imaging on epileptic human neocortical slices removed during epilepsy surgery to show that the activated cortical areas during epileptic activity are focal and their spatial positions change in a dynamic manner within the epileptic tissue. In line with these results, many studies used the size of the area producing interictal epileptic spikes, the irritative zone, as an indicator of the focality and the chance of seizure freedom after surgery [5], [17], [45]-[47]. For this purpose, usually each single spike is localized separately and then the scatter is calculated from the distance of each spike localization to the centroid location. While this approach seems reasonable for high SNRs, it was shown in Bast et al. [2] that the scatter size depends highly on the SNR for EEG single spike localizations. On the other hand, it is possible to average epileptic spikes with similar origins (having a sufficiently similar EEG/MEG topography) to improve SNR and to achieve more reliable source reconstructions [2], [11]. However, the latter approach does no longer provide much information on the actual size of the underlying irritative zone, because it often uses a single dipole that only represents the center of gravity of a larger activated cortical patch. At first view, distributed source models and current density approaches might seem more appropriate, but the reconstructed extent in commonly used current density approaches mainly depends on the chosen approach/norm and huge differences in spatial dispersion have been shown for one and the same underlying source [48]. Here, we propose the concept of subaveraging with the aim of accurately reconstructing the center of gravity and at the same time not losing all information about the possible extent of the irritative zone. One further main topic investigated here is thus assessing the sensitivity of single spike localizations on SNR in order to better estimate both the center and the spatial distribution of the epileptic activity. We used source reconstructions of EEG, MEG and EMEG to outline their specific performance. Multiple subaverages with different numbers of spikes were produced with a bootstrap like algorithm and these subaverages were compared with each other and with single spike reconstructions.

### Structure of our study and main contributions

The remainder of our study is structured in the following way: In the Patient and Methods section we present the medical history of the patient, the construction of the individual FE head model and the source reconstruction procedure including epileptic spike detection and subaveraging. Results and Discussion sections are both divided into the following two subsections: In subsection “Effects of Epileptic Spike Averaging on Source Reconstruction” we present and discuss the differences between different subaverages on the calculated center of gravity and extent of the scatter. In subsection “Comparison of EEG, MEG and EMEG Source Reconstructions” we use our findings on subaveraging in order to assess the differences between EEG, MEG and EMEG source reconstructions during different phases from spike onset to later propagation at the spike peak. Our results in the latter subsection confirm our hypotheses that, unlike single modality EEG or MEG alone, combined EMEG in calibrated multi-compartment realistic head models allows meaningful source reconstructions at early instants in time, i.e., at time points with low SNR (spike onset). These early time points are not yet subject to propagation and thus closer to the origin of the epileptic activity. Furthermore, we show that combined EMEG source analysis reveals the propagation pathways at later time points in complete agreement to sEEG, while single modality EEG or MEG might only be sensitive to, however, complementary, parts of the epileptic activity.

## Patient and Methods

### Ethics Statement

The patient and her parent signed written consent forms and all procedures have been approved by the ethics committee of the University of Erlangen, Faculty of Medicine on 10.05.2011 (Ref. No. 4453).

### Patient

A 17 years old female suffering from pharmaco-resistant focal epilepsy since the age of six has been investigated. In one of the 3T MRIs the radiologist made a remark of a suspected left temporo-mesial FCD that could not be confirmed in later MRI investigations. An FDG-PET scan showed a diffuse and extended left fronto-temporal hypometabolism. The video-EEG (surface EEG) recorded seizures with temporal and frontal semiology and the EEG showed early temporal left and bifrontal/frontal left seizure activity. One of the hypotheses before the invasive EEG was that, while there are several interictal spike foci, there might be an initial temporal left onset with propagation to frontal areas. However, the invasive EEG showed that this is not true, but instead there were three seizure onset zones: frontal basal mesial, rostral frontal and temporal. Resective epilepsy surgery was refused after the invasive work-up due to multifocal activity and an unfavorable risk-benefit ratio of any resective surgical intervention.

### MRI Measurements

T1-weighted (T1w), T2-weighted (T2w) and diffusion-tensor (DT) MRI scans were acquired with a 3T scanner (Gyroscan Intera/Achieva 3.0T, System Release 2.5 (Philips, Best, NL)). Additionally, a diffusion weighted data set with flat diffusion gradients but with reversed encoding gradients was measured to be used for susceptibility correction [49]. T1w and T2w MRIs had 1.17 mm and DT-MRI had 1.875 mm edge length for the measured cubic voxels. MR images were resampled to 1 mm isotropic resolution, used as the resolution of the FE mesh throughout this study. The total acquisition time required for these four scans was 28 minutes.

### Head Model Construction

FSL (http://www.fmrib.ox.ac.uk/fsl) routines-FLIRT and-BETSURF were used to register T1w and T2w MRIs, and to distinguish between scalp (which includes all extracranial tissues), skull and brain. The results of the automatic segmentation algorithm were afterwards inspected and manually corrected. FSL-FAST [50] and Freesurfer (http://surfer.nmr.mgh.harvard.edu/) were later used to distinguish CSF, white and gray matter. The skull spongiosa-compacta classification was implemented by eroding the skull estimate and performing a threshold based segmentation on the T2w MRI (limited to the skull estimate).

The information on anisotropy was included to the white matter using the DT-MRIs. FSL-FLIRT was used for eddy current correction and registration of the DT MRI to T2w MRI. The two data sets with flat diffusion gradients but with reversed encoding gradients were used for susceptibility correction using a diffeomorphic nonlinear correction approach with a variational multiscale nonlinear image registration framework as implemented in the freely-available SPM (http://www.diffusiontools.com/documentation/hysco.html) and FAIR (http://www.mic.uni-luebeck.de/people/jan-modersitzki/software/fair.html) software packages [49]. Following the susceptibility correction, diffusion tensors were determined with FSL-DTIFIT [51]. As a last step, the conductivity tensors were calculated from the artifact-corrected and registered diffusion tensors using an effective medium approach as described in [37], [38].

A geometry adapted hexahedral FE mesh, which was created by shifting the nodes on material interfaces to increase the conformance to the real geometry and to mitigate the staircase effects [52], was constructed based on the labeled MRI using SimBio-VGRID (http://www.rheinahrcampus.de/~medsim/vgrid/index.html) [53]. The overall construction of the head model took about two days, most of it for the manual correction and optimization of the automatic segmentation results.

### Simultaneous EEG/MEG Measurements

80 channel EEG, 275 channel whole head MEG (plus 29 reference channels for synthetic gradiometers) (CTF, VSM MedTech Ltd.) and electrocardiography (ECG) were measured simultaneously in a magnetically shielded room. The amplifiers for EEG system were supplied with the MEG and use the same system clock to ensure synchrony. The EEG cap had 74 Ag/AgCl sintered ring electrodes placed equidistantly according to the 10–10 System (EASYCAP GmbH, Herrsching, Germany). In addition to those 74 electrodes, 6 additional channels were available and used for both eye movement detection (with a bipolar software montage) and as additional EEG channels for source reconstruction. Electrode locations were digitized with a Polhemus Fastrak system (Polhemus Incorporated, Colchester, Vermont, U.S.A.) prior to measurement. The patient was measured in lying position to reduce head movements and to avoid erroneous CSF effects due to brain shift when combining EEG/MEG and MRI [54]. Six runs were acquired in total. The first run was 7 minutes long with the aim to measure somatosensory evoked responses following electrical stimulation of the left median nerve for head model calibration. The next 5 runs (8 minutes each) were spontaneous measurements without any stimulation for spike detection. During the measurements the position of the head inside the MEG scanner was constantly measured via three coils that are placed on nasion, left ear and right ear canal and only the runs with maximum head movement lower than 8 mm were used in further analysis.

### Epileptic Spike Detection

**Fig 1:**
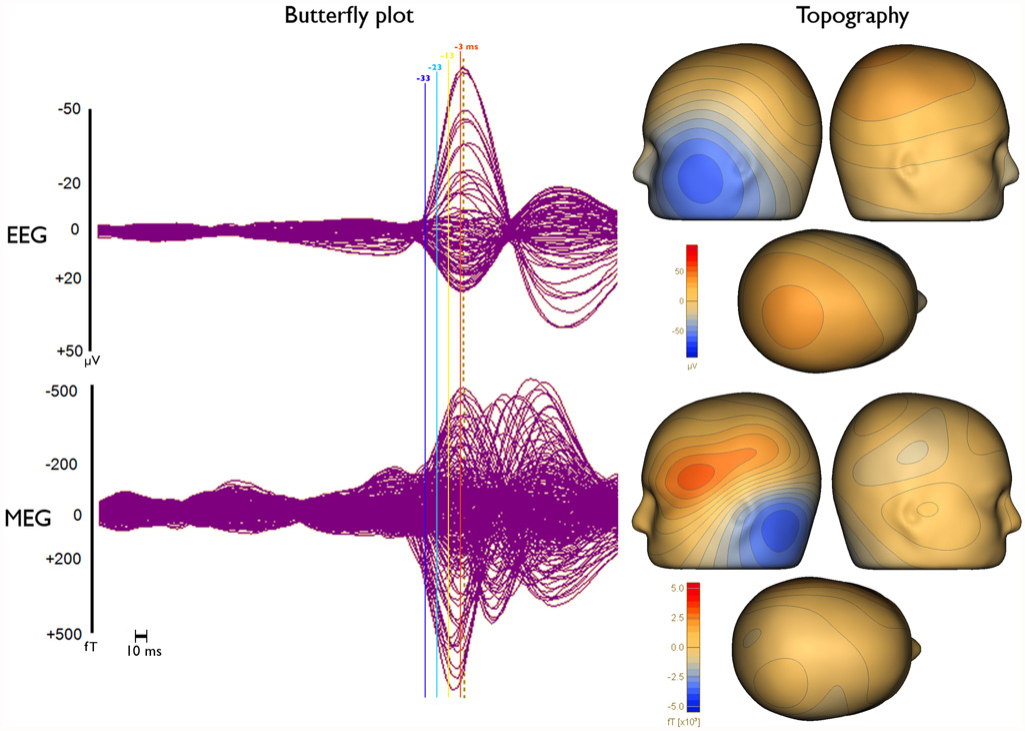
Butterfly plots and topographies of grand-average EEG and MEG spikes. Butterfly plots (left) and topographies (right) of EEG (upper row) and MEG (lower row) for the grand-average (average over all 200 single epileptic spikes). The time points that are discussed in the manuscript, namely -33 ms (dark blue), -23 ms (light blue), -13 ms (yellow) and -3 ms (orange), are indicated by solid vertical lines with different colors. Topographies are shown for the EEG spike peak (time point 0 ms) as indicated by the dashed vertical line in the butterfly plots.

As a first step the 3 runs with minimal head movement were filtered with an 80 Hz low pass filter, resampled to 250 Hz, concatenated, and corrected for ECG artifacts using BESA (http://www.besa.de). For this purpose, the ECG channel was selected for detection and averaging, and principal component analysis was used for minimizing the artifact. The measurements were then examined and epileptic spikes were marked by 3 clinical reviewers (PK, CK, SR). From these markings, 10 clear left temporal spikes, which belong to the most frequent spike type, were selected using source montage (temporal) and averaged. The averaged spike was used in BESA template search in order to find further spike candidates. Selecting left temporal polar, basal and lateral channels from the temporal source montage for template search ensured the detection of spikes with similar location and orientation. After visual inspection 200 left temporal spikes without any clear artifacts (e.g., eye-blinks), were selected for further analysis. The butterfly plots of the grand-averaged (all 200 spikes) EEG and MEG signals, 4 solid vertical lines with different colors indicating the important instants in time, namely -33, -23, -13 and -3 ms, which are discussed in the manuscript, and the respective topographies at 0 ms (signal peak in EEG shown with dashed vertical line) after averaging are visualized in Fig. 1.

### Subaveraging

Ten subaverage groups each with 200 realizations were constructed from the 200 single spikes detected in the previous section. Each realization in each subaverage group was an averaged signal that was calculated using the same number of single spikes and the name of the subaverage group was determined by this number. For example in the Av10 subaverage group there were 200 Av10 realizations, each of them obtained by random drawing and averaging 10 single spikes. The random drawing was performed with Matlab (The Mathworks, Inc.) and it was ensured that none of the spikes was chosen more than once in the same realization. The subaverage groups that were constructed with this procedure were Av5, Av10, …, Av50 with increments of 5. Additionally, the group containing all 200 single spikes was denoted Av1.

The continuous EEG and MEG data were imported into Curry (http://www.neuroscan.com/curry.cfm), filtered from 1 to 100 Hz and divided into 400 ms long epochs (200 ms before and 200 ms after each EEG spike peak). The SNRs were calculated by dividing the signal power at a certain time point, which was used for source reconstruction, by the variance of the noise determined from the interval -200 to -70 ms. The SNRs of EEG, MEG and EMEG signals were calculated separately for each time instant (from -33ms to 0 ms) and only the signals with SNRs higher than three in the corresponding modality and subaverage were included in the further analysis.

### Source Reconstruction Procedure

A cortically-constrained deviation scan inverse approach was used for source reconstruction [55], [56]. For this purpose, a source space with 2 mm resolution was calculated. A custom written Matlab code ensured that all source space points were located inside the gray matter compartment and sufficiently far away from other tissues. This ensured the closest node of the FE mesh for each source space point to only belong to elements that were labeled as gray matter. Thus, the so-called Venant condition, being crucial to avoid unrealistic source modeling when using the FE-Venant approach, was satisfied [57]. The SimBio software (https://www.mrt.uni-jena.de/simbio and the SimBio integration into Fieldtrip: http://fieldtrip.fcdonders.nl/development/simbio) was then used to calculate EEG and MEG leadfield matrices for the given source space, FE head model and sensor configurations. The Venant source modeling approach was selected together with standard piecewise trilinear basis functions and an FE transfer matrix approach using an algebraic multigrid preconditioned conjugate gradient solver to obtain numerically accurate and computationally efficient forward EEG and MEG solutions [53], [57], [58]. The calculation of the leadfield matrix for 80 channels EEG and 275 channel MEG took about 60 minutes and 6 hours respectively on a standard workstation (Intel Core i7-860 Processor, 2.80 GHz and 16 GB RAM). The leadfield matrices were then imported into Curry and single dipole scans were calculated from -33 to 0 ms with 0 corresponding to the peak of the EEG signal. Unlike classical dipole fit algorithms, the cortically-constraint deviation scan provides goodness-of-fit (GOF) values for all source space points and the location with the highest GOF was then used as the final deviation scan result.

Unlike MEG source reconstructions in which we used regularization to avoid implausible results, which might occur due to MEG’s insensitivity to radial components, for EEG and EMEG we did not use any regularization. Furthermore, only the MEG sensors over the left hemisphere were used for MEG and EMEG source reconstructions to improve SNR and GOF.

In order to perform combined source reconstruction, EEG and MEG signals were transferred to a unitless common space. This was achieved by using the SNRs of each electrode and gradiometer instead of the original signals [25].

In the results section we also use, instead of spike clusters, so-called centroid dipoles, defined by the mean location and orientation of all deviation scan result dipoles belonging to the corresponding cluster. In addition, in spike clusters the distances of each deviation scan reconstruction (that passes the SNR criterion) to the centroid dipole were determined and used to calculate the mean distance and its standard deviation for each cluster. Then, the reconstructions in which the distance to the centroid exceeds mean plus two times the standard deviation for this cluster were excluded from the cluster and not used for further analysis (see Algorithm 1 in [40] for details).

### Skull Conductivity Calibration

EEG and, to a lesser extent, EMEG source reconstructions are sensitive to the conductivities of the highly isolating skull tissues [25], [40], [42]. However, skull conductivity values are quite controversial in literature with huge interindividual variance [26], [33], [59], [60]. We therefore calibrated the skull conductivity using the N20 component of the measured and reconstructed somatosensory evoked potential (SEP) and field (SEF) data as described and evaluated in detail in [40]. Similar calibration procedures have also been proposed by [41], [42].

The tissue conductivities (S/m) that are used in this study are [40], [61], [62]: Scalp (0.43), CSF (1.79), gray matter (0.33), anisotropic white matter along with the skull conductivity values individually calibrated for the patient: skull compacta (0.0024) and skull spongiosa (0.0084).

### Stereo-EEG Measurements

**Fig 2:**
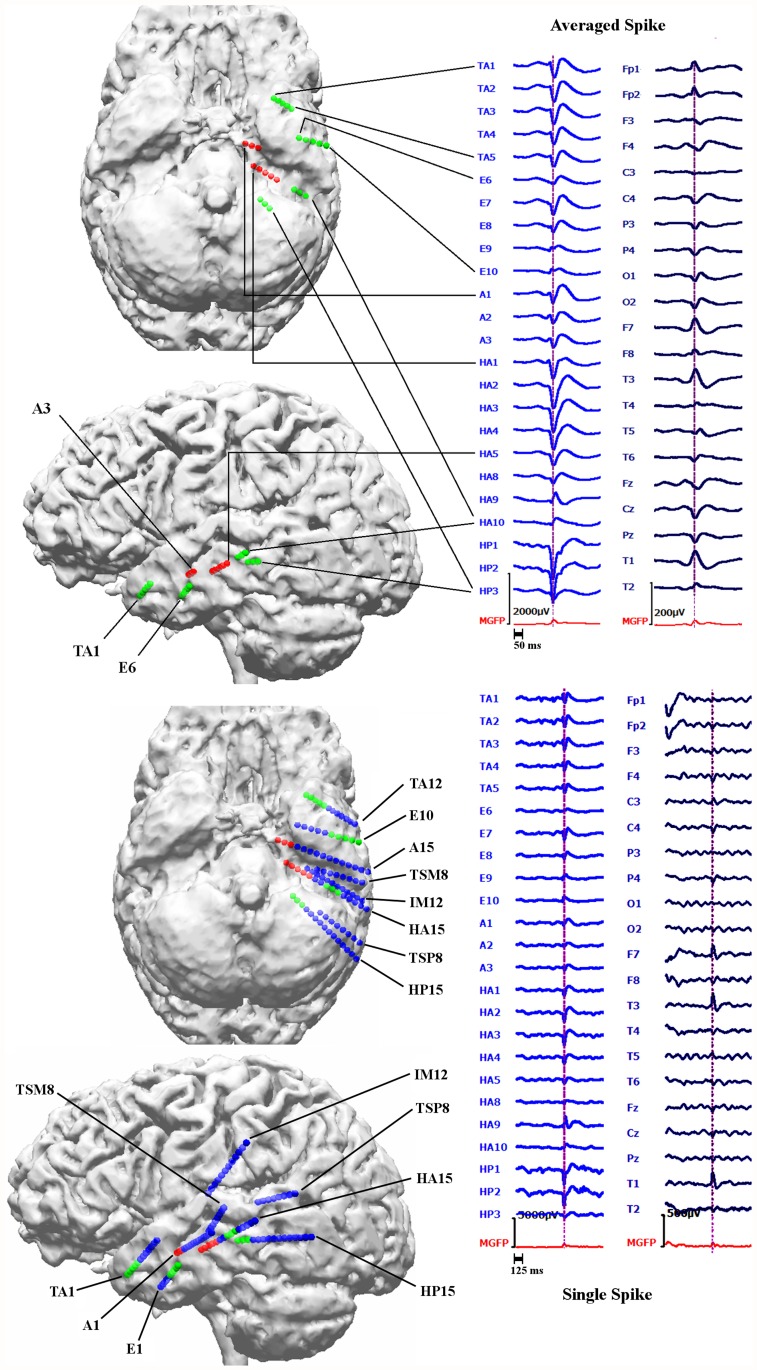
Locations of sEEG contacts inside the brain and epileptic activity measured with sEEG and ldEEG. Examples for an averaged (top right block) and a single epileptic spike (bottom right block) measured simultaneously with sEEG (left column in each block) and 21 electrodes low density scalp EEG (ldEEG) (right column in each block). Maximum of sEEG is at the HP2 contact as shown by the vertical lines at the peak of this contact. In the upper two brain figures (left block) only the active (that measures interictal or ictal signals) sEEG contacts are indicated while the bottom two indicate the positions of both active and not active sEEG contacts close to the left temporal lobe. The union of green and red spheres shows the sEEG contacts measuring frequent interictal activity, red spheres alone show the contacts measuring seizure onset and blue contacts do not measure interictal or seizure activity.

sEEG relying on 14 intracerebral depth electrodes with 167 contacts in total, showed independent epileptic activity with left frontal and temporal origins from 3 different seizure onset zones: i) left fronto-basal mesial, ii) left temporal and iii) left frontal parasagittal. In this paper we focused only on the left temporal spikes due to their high occurrence rate. 24 contacts from 5 different electrodes and 8 contacts from 2 electrodes were active during temporal interictal spikes and seizure onset, respectively. Examples of an averaged spike and a single spike measured simultaneously with sEEG and low density scalp EEG (ldEEG) (21 electrodes) are shown in Fig. 2. Based on information from the clinical report we represent the sEEG contacts with three different colors: The green spheres represent the sEEG contacts that measured only interictal activity, red spheres represent the sEEG contacts active during the seizure onset and the blue spheres represent the contacts near the left temporal lobe that measured neither interictal nor seizure onset activity. All contacts that were active during seizure onset were also measuring interictal spikes and were thus within the irritative zone. The locations of the sEEG contacts were marked manually using the post-operative T1-MRI and computed tomography images that had been registered to the pre-operative T1-MRI using an affine registration scheme.

### Square Distance Index (SDI)

In order to quantify the amount of agreement between noninvasive source reconstructions and sEEG, we used a formula which weights each dipole by the inverse of its square distance to each active sEEG contact.

$$SDI_{i}=\frac{\sum_{j=1}^{N}\frac{1}{d_{j}^{2}+1}}{N}\times 100$$ 1

Where *i* represents a specific sEEG contact, e.g., A1, which measures frequent interictal activity, *N* is the number of dipoles that passes the SNR criterion (SNR>3) for the respective modality and *d*_*j*_ represents the Euclidian distance between the j’th dipole and the sEEG contact i. The addition of 1 ensures the appropriate weighting for perfect localizations (*d*_*j*_ = 0). A high value of this index at a certain sEEG contact indicates concentrated dipole localizations in the vicinity of this contact. On the other hand, high differences in SDIs for different sEEG contacts indicate that the localizations highlight only a certain area within the irritative zone and not the whole. Thus, the mean of SDIs over all sEEG contacts should be high, while its standard deviation over different contacts should be low for an accurate and complete depiction of the irritative zone.

## Results

Our result section is divided into two subsections. In the first subsection, we investigate epileptic spike subaveraging with the aim of finding the subaverage number that allows both an appropriate reconstruction of the center of gravity and an estimation of the size of the irritative zone. The determined optimal subaverage number is then used in subsection two to investigate sensitivity differences of EEG and MEG, and especially to evaluate the contribution of combined EEG/MEG in comparison to single modality EEG or MEG source analysis of the epileptic activity.

### Effects of Epileptic Spike Averaging on Source Reconstruction

Fig. 1 (left column) presents butterfly plots of the grand-averaged (all 200 spikes) signals in EEG (upper row) and MEG (lower row). As the dashed vertical line in this figure clearly shows, the MEG signal peak (time point-7 ms) precedes the EEG signal peak (time point 0 ms) by 7 ms. Furthermore, the time instants that are discussed in the following are indicated in Fig. 1 by solid vertical lines with different colors (-33 ms in dark blue, -23 ms in light blue, -13 ms in yellow and -3 ms in orange).

**Fig 3:**
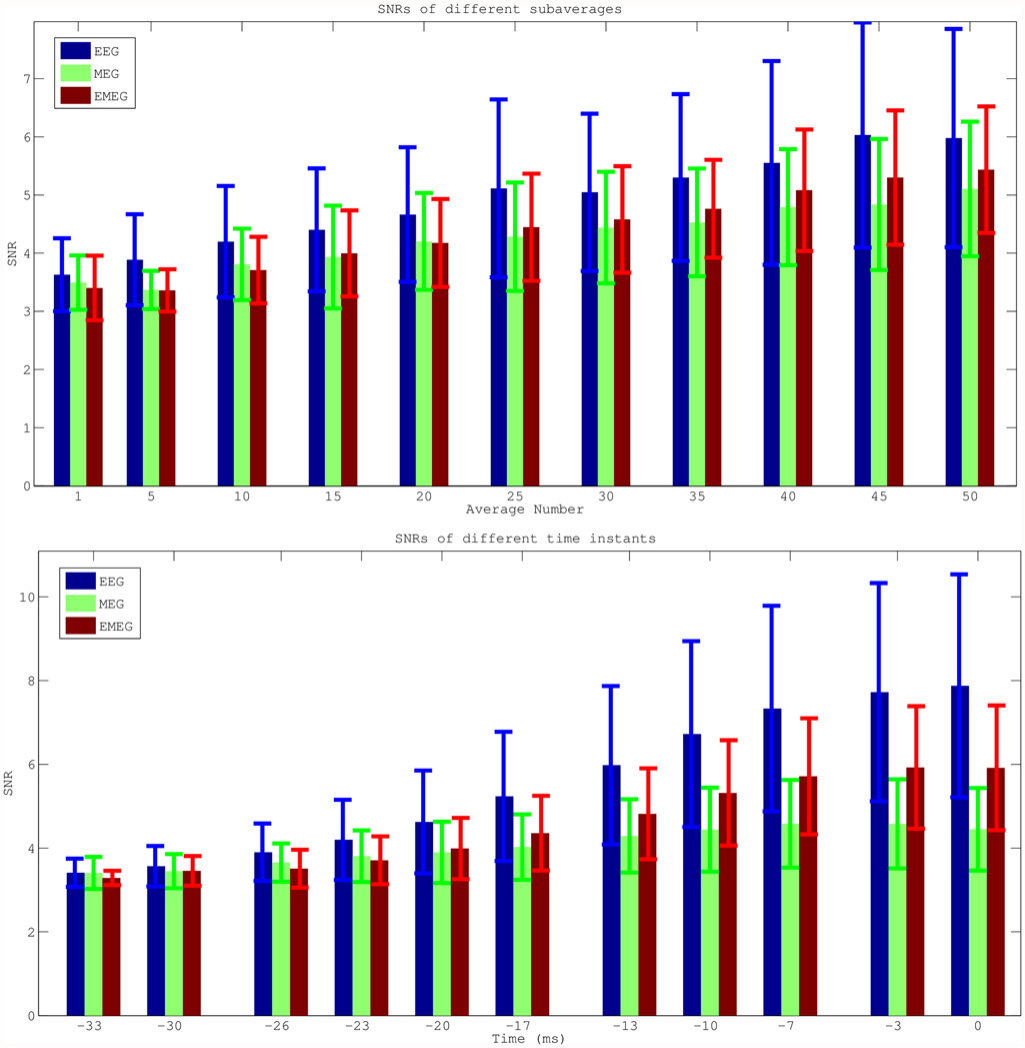
Mean SNRs for EEG, MEG and EMEG for different subaverages and different phases. Average SNRs (with error bars indicating the standard deviations) for EEG (blue), MEG (green) and EMEG (red). Only single epileptic spikes (Av1) or subaverages (Av5-Av50) with SNRs higher than three have been taken into account in these figures. The upper row shows the results for different average numbers for time point-23 ms at the rising flank of the averaged epileptic spike and the lower row for different time points from -33ms (spike onset) to 0ms (spike peak in EEG) for subaverage 10 (Av10).

Next, the SNRs of the different modalities, subaverages and time points were investigated. Fig. 3 shows the SNR results (average values with error bars indicating the standard deviations) for EEG (blue), MEG (green) and EMEG (red). The upper row presents the results for time point-23ms at the rising flank of the averaged spike (see light blue vertical line in Fig. 1) for different average numbers. It shows only single spikes with a minimal SNR of 3 (Av1) or subaverages (Av5 to Av50) made up of spikes with possibly lower SNRs than 3, but which then reach the threshold of 3 within the averaging procedure. As a result of this, and because the amplitudes of the single spikes vary, the SNR does not increase with the square-root of the average number of spikes as it would be expected for example in an analysis of evoked responses. However, it still clearly increases with increasing average number and for all three modalities. Fig. 3 (lower row) shows the results for subaverages of 10 (Av10) for different time points. A different behavior of EEG and MEG over time can be observed in this subfigure. At spike onset (time point -33 ms, see dark blue vertical line in Fig. 1), EEG and MEG SNRs are almost identical. However, in later instants in time at the rising flank of the epileptic spike (time points -30 ms to -3 ms) the SNR of the EEG increases faster than the SNR of the MEG, which leads to considerable differences in SNRs at the EEG spike peak (time point 0 ms). Finally, it is also clearly visible that the standard deviations (error bars) increase with increasing SNR.

**Fig 4:**
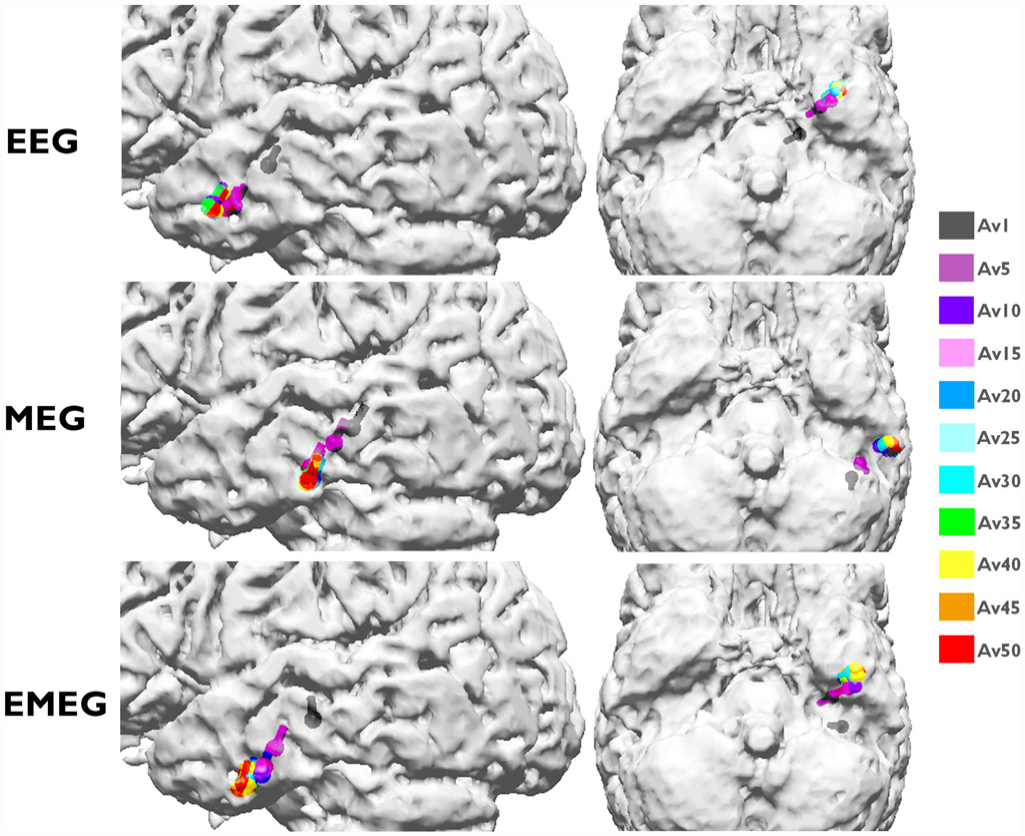
Centroid dipoles of EEG, MEG and EMEG for different subaverages at -23 ms. Centroid dipoles determined from the dipole scan peaks of EEG (upper row), MEG (middle row) and EMEG (lower row) for different subaverages at 23 ms before the EEG spike peak. Each color shows centroids for different subaverages and Av1 is the centroid for single spike reconstructions. The centroid dipoles were computed from those random realizations out of 200 random realizations for each group, which satisfied the SNR>3 criterion.

We then calculated centroid dipoles for different subaverages at -23 ms (see light blue vertical line in Fig. 1) and visualized the results in Fig. 4. This figure shows large differences in source reconstructions between single and subaveraged epileptic spikes. The single spike source reconstructions (Av1) are considerably more superior and deeper than the subaverages for EEG, MEG and EMEG indicating a systematic noise bias of Av1. The noise bias in Av5 has a similar tendency, but is already much smaller than for Av1, and no more bias can be observed for subaverages with more spikes, especially for MEG and EMEG. Starting from Av10 and further increasing the number of averaged spikes results in only minor changes to the centroid dipoles. Fig. 4 thus shows that a minimal average number of 10 should be used for this patient to avoid a noise bias in the reconstruction of the center of gravity of the irritative zone.

**Fig 5:**
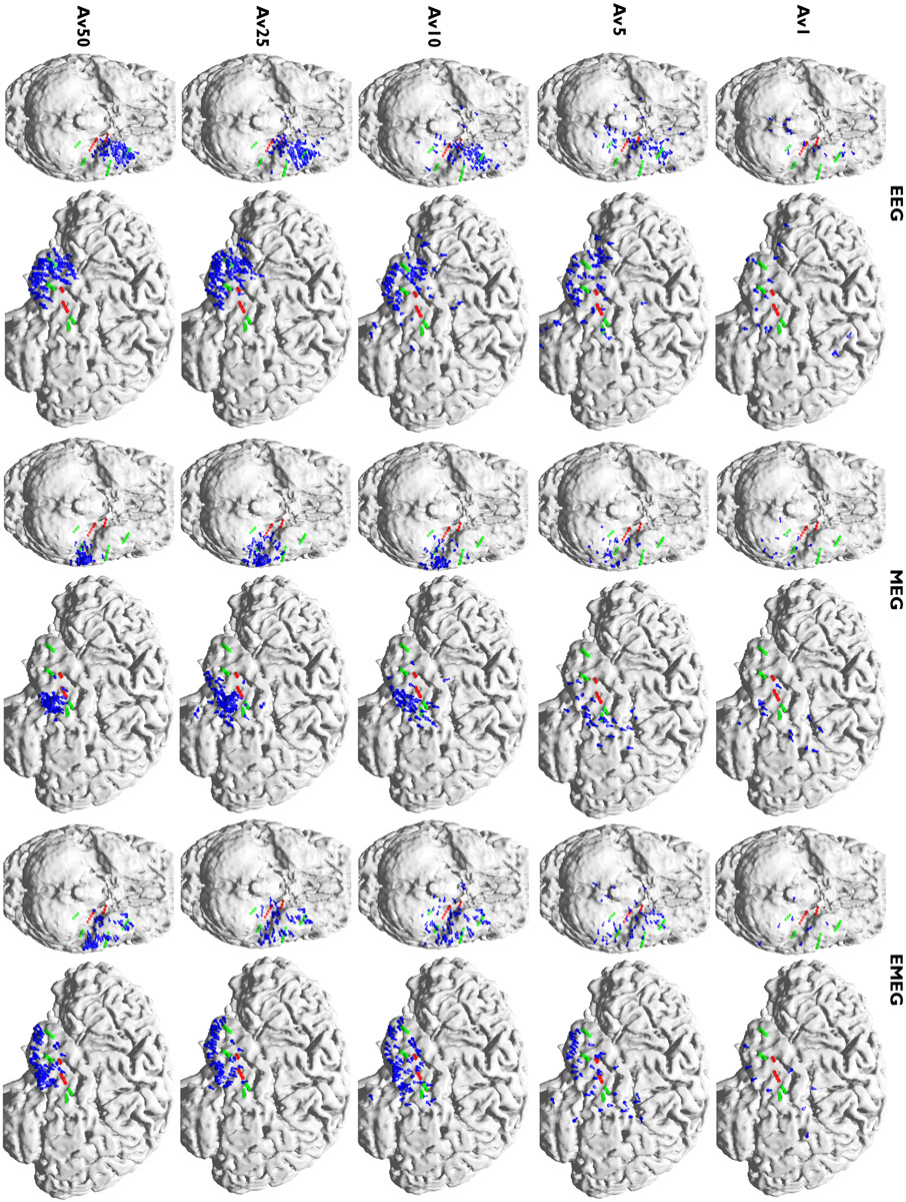
Peaks of the deviation scans of EEG, MEG and EMEG for different subaverages. Peaks of the deviation scans (illustrated by blue dipoles) of EEG (left two columns), MEG (middle two columns) and EMEG (right two columns) for different subaverages. The figure shows the results at 23 ms before the peak of the EEG. Both green and red spheres show the sEEG contacts where frequent interictal activity can be measured, thus giving an impression of the minimal size of the irritative zone, and red spheres alone show seizure onset contacts.

Fig. 5, which shows, visualized with blue dipoles, the deviation scan peaks for Av1, Av5, Av10, Av 25 and Av50 at -23 ms, further strengthens and complements the results of Fig. 4. Green and red spheres in Fig. 5 represent the sEEG contacts which measured frequent interictal epileptic activity, thus giving an impression of the minimal size of the irritative zone, and red spheres are the sEEG contacts that additionally measured ictal activity. Most of the observations in this figure are similar for EEG, MEG and EMEG, and as long as the modality name is not explicitly mentioned in the following description, these observations are valid for all three of them. It is clear from Fig. 5 that very few single spikes (Av1) pass the SNR criterion (SNR>3) and even among them spurious dipoles (outliers) persist. When the subaverage number is increased to five, although the localizations become more stable and dipole clusters start to emerge, still many outliers can be observed. For Av10 the clusters become more distinguishable with only few outliers. These results thus support our argumentation from the previous paragraph (Fig. 4) to use a minimal average number of 10 for this patient. The following argumentation now delivers the complementary information that, from the chosen subaverage numbers, Av10 even seems to be optimal: Av25 results again differ from Av10, especially with regard to a further decrease in the scatter of dipoles. When increasing the subaverage number to 50 the scatter of the dipoles further decreases and MEG epileptic spikes are localized more lateral than for Av10 and Av25. Now the dipole scatters can be evaluated with the information from the sEEG. Fig. 5 shows that for Av10 the dipole scatter covers almost all active sEEG contacts, i.e., it covers the minimal size of the irritative zone. For Av25 the clusters are already too focal, missing the active HA8–10 and all HP contacts. Av50 is even more focal, missing even more of the active sEEG contacts (additionally to HA8–10 and all HP, also HA1–5 are outside the estimated irritative zone) and thus strongly underestimating the size of the irritative zone.

### Comparison of EEG, MEG and EMEG Source Reconstructions

For comparing EEG, MEG and EMEG localizations, based on the results of the previous subsection, the focus will be on Av10 results. This choice is based on Figs. 4 and 5, which show that a minimal average number of 10 is needed to sufficiently reduce noise bias and appropriately reconstruct the center of gravity of the irritative zone and that higher average numbers result in too focal dipole clusters that lead to an underestimation of the extent of the irritative zone.

The Av10 EEG reconstructions in Fig. 5 are mainly localized in an area close to the pole of the temporal lobe and close to sEEG TA contacts (Temporal Anterior, see Fig. 2). On the other hand, no activity is localized near HP1–3 and HA1–5 (the hippocampus posterior and anterior contacts). In MEG the localizations are more posterior than in EEG with clusters in the vicinity of HA8–10 (the posterior lateral neocortical contacts that, in contrast to their label, are not located in the hippocampus anterior, see Fig. 2), and close to HP contacts although no cluster was formed around them. Unlike EEG there are no localizations in the vicinity of TA in MEG. In EMEG, noninvasive reconstructions cover all active sEEG contacts. EMEG even shows localizations in the vicinity of the HP contacts, where neither the sensitivity of EEG (see EEG side-view in the second column of Av10 in Fig. 5) nor of MEG (see MEG bottom-view in the third column of Av10 in Fig. 5) was sufficient to reconstruct any activity.

**Fig 6:**
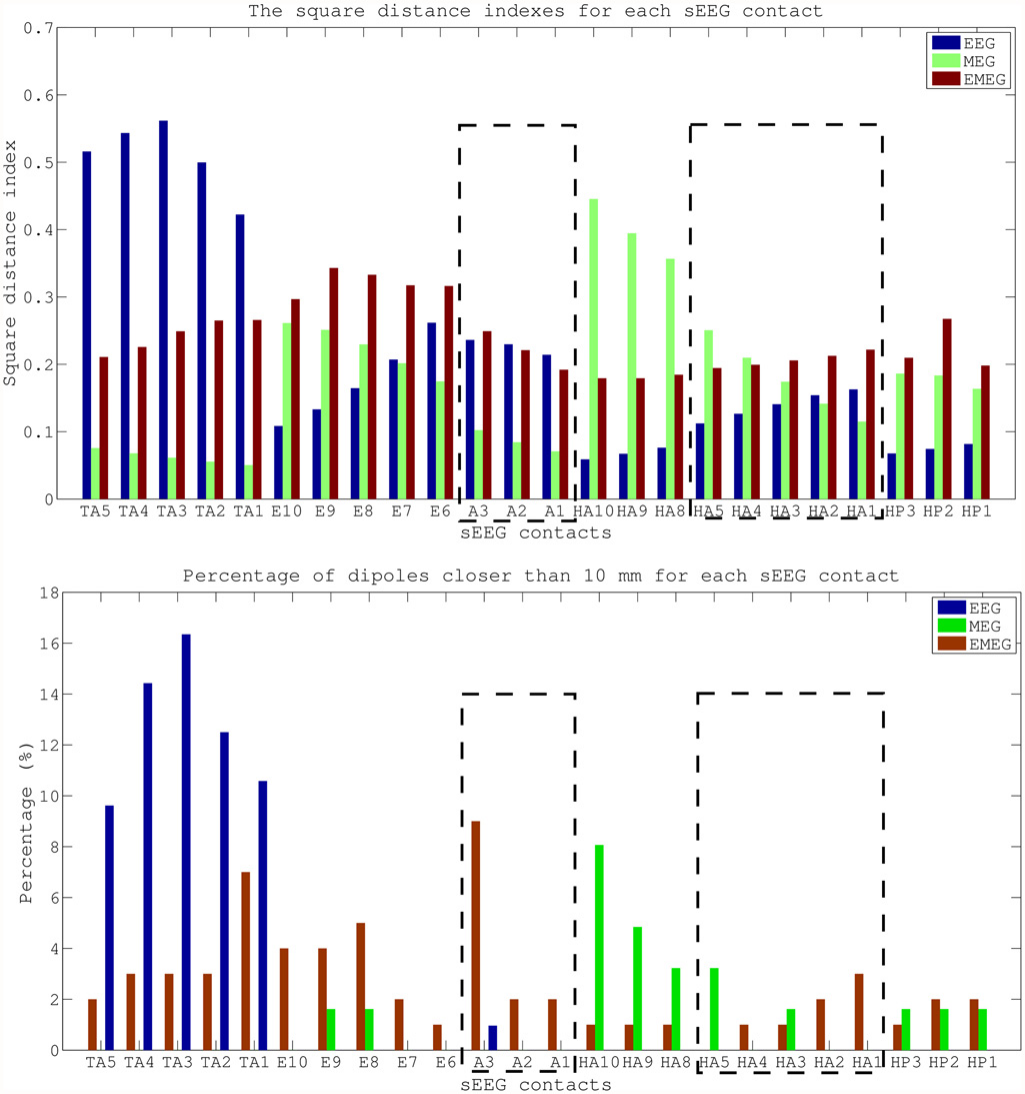
Quantitative comparison of EEG, MEG and EMEG source reconstructions and sEEG contacts. Square distance index and the percentage of dipoles closer than 10 mm for each sEEG contact. The values are given for EEG, MEG and EMEG for subaverages of 10 at -23 ms. The sEEG contacts enclosed by dashed lines are within the seizure onset zone.

The plots in Fig. 6 add quantitative information to Av10 source reconstructions visualized in Fig. 5. The plots show the SDIs (upper subfigure) and the percentage of dipoles that are closer than 10 mm to each sEEG contact measuring frequent interictal activity (lower subfigure). In these plots, the contacts that are also part of the seizure onset zone (amygdala contacts A1–3, and hippocampus anterior contacts HA1–5) are enclosed within rectangles with dotted lines. The upper subfigure clearly shows that most of the EEG localizations are clustered near the TA contacts and the lower subfigure shows that there are dipoles within 10 mm at only 6 out of overall 24 interictal and 1 out of 8 ictal contacts. MEG values for the same measures are 10 out of 24 interictal and 2 out of 8 ictal, and the localizations are clustered especially near the posterior lateral neocortical contacts HA8–10 and HP contacts. On the other hand, for EMEG 23 out of 24 interictal and 7 out of 8 ictal contacts have at least one noninvasive localization within 10 mm. In the SDI plot the EEG SDIs for TA contacts are considerably higher than for other contacts, while for MEG the SDIs at contacts HA8–10 are larger. For EMEG the SDI index is almost equally distributed over the sEEG contacts and does not show a huge variation as in EEG and MEG. The means and standard deviations of SDIs for EEG, MEG and EMEG demonstrate this behavior well. The average SDIs for EEG and MEG are 0.22±0.15 and 0.18±0.11, respectively, and with 0.24 the average SDI for EMEG is higher and, most importantly, with 0.05 its standard deviation is considerably lower than for EEG or MEG.

As already noted, the MEG signal peak precedes the EEG maximum by approximately 7 ms (Fig. 1). In order to investigate which sources are dominating the EEG and MEG signals, we compared the timings of epileptic spikes in sEEG and the simultaneously measured ldEEG (Fig. 2). We observed that simultaneously with the ldEEG peak, sEEG contact TA4 is also at its peak value. On the other hand, the peak of the HP2 contact, the contact which measures the highest amplitude in sEEG, is occurring 7.5 ms before the ldEEG and TA4 peaks. Moreover, the peaks of A1–3 and HA1–5, i.e., the seizure onset contacts, are also preceding the TA contacts and are almost simultaneous with the HP contacts.

**Fig 7:**
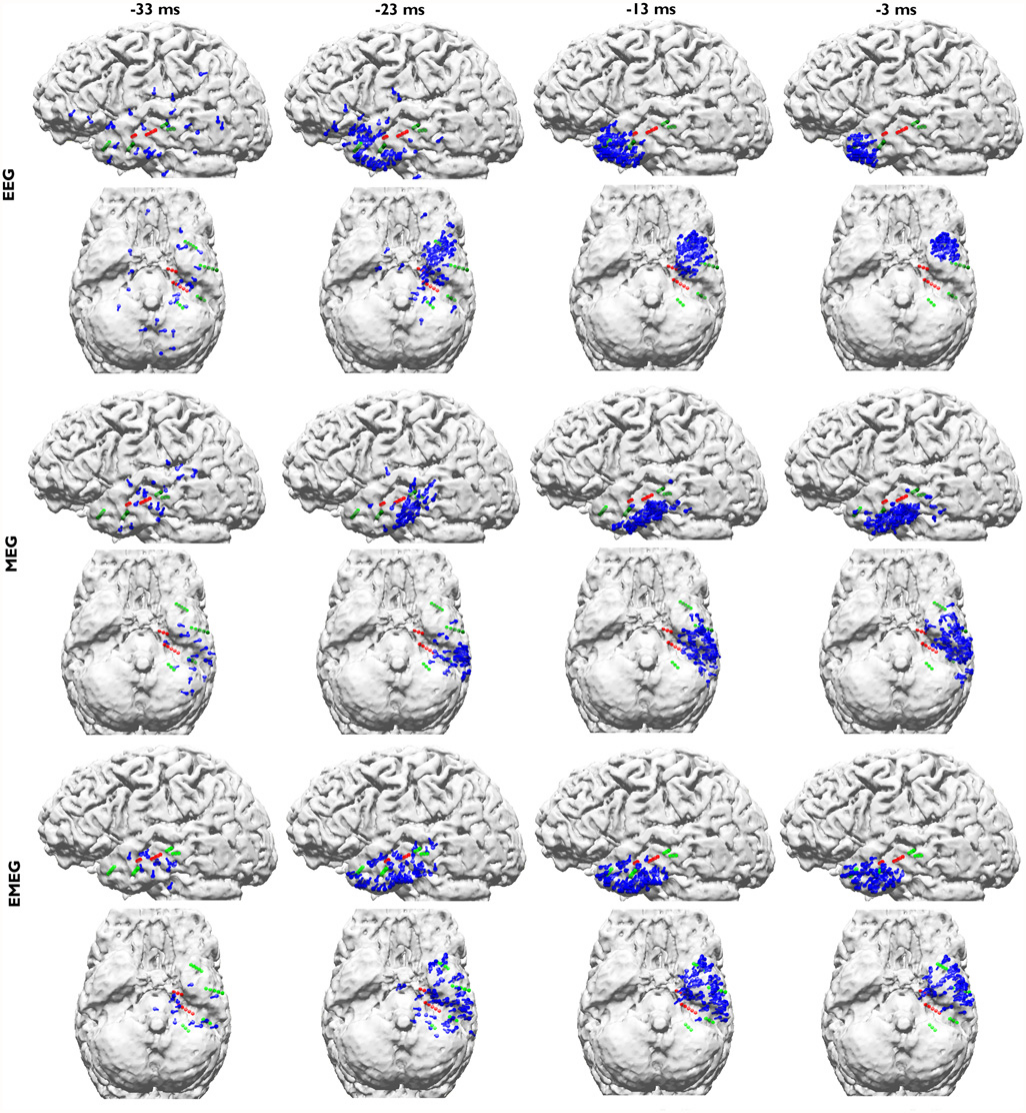
EEG, MEG and EMEG deviation scan peaks of Av10 at different propagation phases. EEG (upper two rows), MEG (middle two rows) and EMEG (lower two rows) deviation scan peaks (illustrated by blue dipoles) of Av10 for time points -33 ms (spike onset, left column) in steps of 10 ms until time point -3 ms (late propagation phase close to EEG peak, right column). Both green and red spheres show the sEEG contacts where frequent interictal activity can be measured, thus giving an impression of the minimal size of the irritative zone, and red spheres alone show seizure onset contacts.

Fig. 7 shows the pathways from spike onset to late propagation determined from EEG (upper two rows), MEG (middle two rows) and EMEG (lower two rows). Av10 deviation scan reconstructions for 4 different time points are visualized from -33 ms (spike onset, left column; also see dark blue vertical line in Fig. 1) to -3 ms (late propagation phase close to EEG peak, right column; also see orange vertical line in Fig. 1) in steps of 10 ms. The first striking observation in this figure is the considerably higher stability of EMEG source reconstructions at spike onset in comparison to single modality EEG and MEG. The EMEG source reconstructions at time point -33 ms are correctly clustered close to the seizure onset zone (red spheres at A1–3 and HA1–5 contacts). In contrast, EEG is strongly dominated by noise with source reconstructions spreading over a wide region. Although MEG reconstructions at spike onset are already better than EEG, they are still too lateral and spread over a too large region and the low SNR still leads to many spurious reconstructions. The results for EEG, MEG and EMEG at the propagation phase also differ between one another. EMEG source reconstructions show that during the 10 ms period from spike onset to time point-23 ms, at the rising flank of the signal, the reconstructed activity spreads from amygdala and hippocampus to a wider area over the temporal lobe, then covering all active sEEG contacts. At time points-13 ms and -3 ms, the reconstructed EMEG activity accumulates near the pole of the temporal lobe. The propagation paths shown by single modality EEG and MEG differ quite much from the one of EMEG and, when compared to the sEEG findings and the EMEG reconstructions, are both incomplete. For EEG, the first stable (by improved SNR) source reconstructions shown in Fig. 7 are the ones at -23 ms in the vicinity of the pole of the temporal lobe. EEG alone completely misses the more posterior activity close to the HA and HP contacts (see Fig. 2) in this early propagation phase (see especially the first row and second column in Fig. 7). At later instants in time the EEG is only able to reconstruct activity at the tip of the temporal pole. With regard to the MEG, at -23 ms, source reconstructions are at more posterior temporal areas covering especially the HA8–10 contacts (see Fig. 2 and second row in Fig. 7) very well. Later on at time points-13 ms and -3 ms, the reconstructed MEG activity travels to more anterior and temporobasal regions. During the whole propagation phase MEG alone completely misses the temporo-polar activity close to the TA contacts (fourth row in Fig. 7).

**Fig 8:**
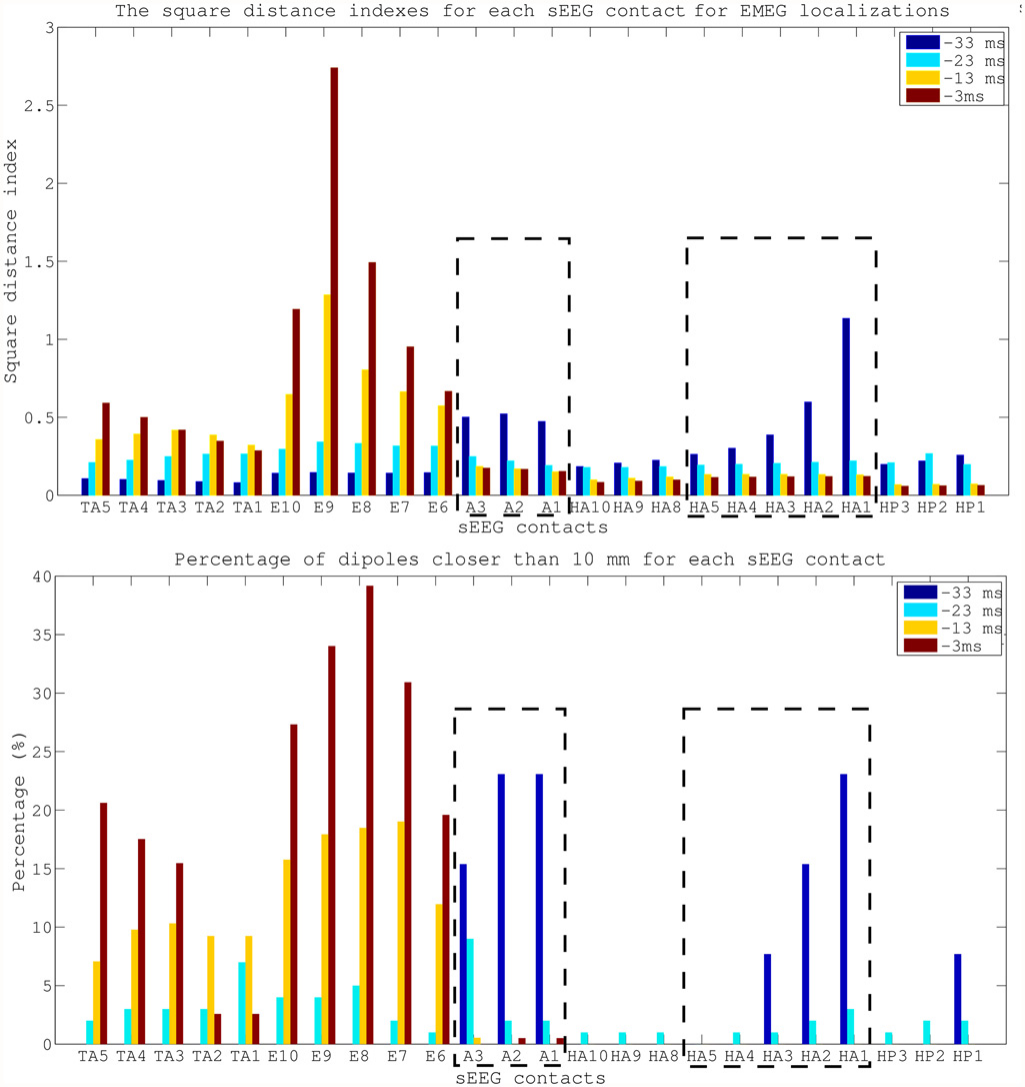
Quantitative comparison of EMEG source reconstructions and sEEG contacts at different propagation phases. Square distance indexes and the percentage of dipoles closer than 10 mm for each sEEG contact. The values are given for Av10 EMEG subaverages at -33, -23, -13 and -3 ms. The sEEG contacts enclosed by dashed lines were within the seizure onset zone.

Corresponding to the visualizations of the propagation pathway in Fig. 7, the plots in Fig. 8 add quantitative information on Av10 EMEG source reconstructions for the 4 different time points (please see supporting information for the same figure for EEG (S1 Fig.) and MEG (S2 Fig.)). The upper subfigure, presenting the SDIs, shows that at -33 ms (spike onset) the source localizations are mostly clustered near the A and HA1–5 contacts (seizure onset). At later time points, i.e., closer to the spike peak, the reconstructed activity propagates to E and TA contacts. The lower subfigure, presenting the percentage of dipoles that are closer than 10 mm to each sEEG contact measuring frequent interictal activity, shows that at -33 ms 7 contacts are covered by the noninvasive EMEG reconstructions. Among them, 6 are ictal contacts (it covers 6 out of 8 ictal contacts) and the other one is the HP1 contact, which peaks earlier than the TA contacts, as shown in Fig. 2. While the EMEG reconstructed activity continuously decreases over time at the seizure onset amygdala and hippocampal contacts, it continuously increases at most of the TA and E contacts.

**Fig 9:**
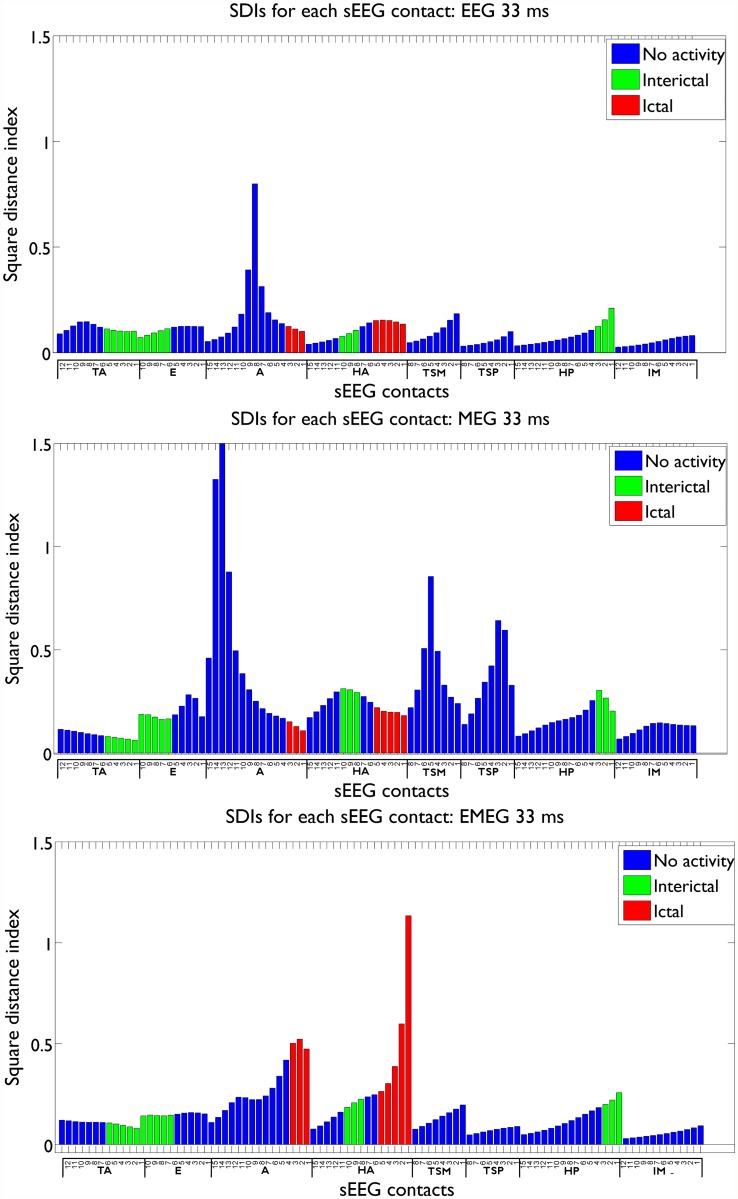
SDI values for EEG, MEG and EMEG at -33 ms. The SDI bars are colored according to the measured activity: seizure onset contacts (red), only interictal contacts (green) and not active contacts (blue).

In Figs. 6 and 8 we compared the SDI values on active sEEG contacts to study the sensitivity of our noninvasive source reconstruction results. For studying specificity, we plotted in Fig. 9 the SDI values for EEG, MEG and EMEG at -33 ms, now also including the not active sEEG contacts. The SDIs were colored according to the measured activity: seizure onset contacts (red), interictal contacts (green) and not active contacts (blue). The figure shows that not only the sensitivity but also the specificity of EMEG results are superior to EEG and MEG alone. EMEG SDIs of sEEG contacts are gradually decreasing with distance to the seizure onset contacts (see HA6 to HA15 and A4 to A9). EEG and MEG source reconstructions alone not only failed to highlight the seizure onset due to low SNRs but also their specificities were qualitatively inferior to our EMEG results.

## Discussion

This section starts with the most important results first, the ordering of the subchapters was therefore inverted.

### Comparison of EEG, MEG and EMEG Source Reconstructions

**Propagation phenomenon: Problems and opportunities for noninvasive source reconstruction**. Propagation of interictal epileptic activity is a well-known phenomenon which might lead to misinterpretations and spurious diagnosis if not taken into account. In order to cope with it, many studies suggested reconstructing sources at the middle of the rising flank instead of the peak of the epileptic spike (see [63] and references therein). Although this might seem to be a good compromise between low SNRs at the spike onset and propagation at the spike peak, the activity at the middle of the rising flank might have already been subject to propagation, e.g., in mesial temporal lobe epilepsy, as shown in this study. On the other side, in cases where the propagation pathway is always identical over different spikes, like the case discussed here, propagation provides also a great opportunity: Propagation of activity from low SNR locations (in our case the deep amygdala and hippocampal structures) to locations with much higher SNRs (in our case the pole of the temporal lobe; see Fig. 3 with regard to the increase in SNR) enables the examiner to find spikes and thus supply the necessary triggers for averaging, which in turn might then enable revealing the preceding activity with lower SNR. This has been shown in our study where we were able to accurately (with regard to our sEEG validation measure, see further discussion below) reconstruct the complete pathway of the epileptic activity from onset to spike peak using subaveraging techniques and combined EEG and MEG source analysis. Similar scenarios are discussed in the literature. For example, simultaneous scalp and intracranial EEG studies showed that, while scalp EEG might not directly be able to distinguish the activity from deeper structures from the noise, it might be possible to extract the EEG signal by averaging intracranial epileptic spikes [64], [65].

**EEG, MEG and EMEG source reconstructions at spike onset**. Despite the strategies explained in the previous paragraph, as we have shown in Figs. 7 and 8, at very early instants in time (-33 ms, see dark blue vertical line in Fig. 1) single modality EEG or MEG source reconstructions might not be reliable enough to draw conclusions on the origin of the epileptic spike. In our results, EEG was strongly dominated by noise and although MEG source reconstructions were more stable, they had a lateral bias with still too many spurious dipole positions. The source reconstructions with single modality EEG or MEG stabilized at later instants in time (see time point-23 ms in Fig. 7), but the activity had already been subject to propagation by then. One of the most important and clinically relevant findings of this study is thus the ability of EMEG to benefit from the complementary information of EEG and MEG at especially these very early instants in time and to thereby stabilize the source reconstructions in cases of low SNR. As shown in Figs. 7 and 8, at -33 ms the source reconstructions of EMEG are mainly clustered near amygdala and hippocampus, i.e., regions within the seizure onset zone as estimated from sEEG.

**Differences of EEG, MEG and EMEG source reconstructions in revealing the propagation pathway**. The EEG, MEG and EMEG source reconstructions differed not only at spike onset but also at later instants in time. At -23 ms (see light blue vertical line in Fig. 1), while both EEG and MEG source reconstructions were clustered in the vicinity of spiking sEEG contacts, they highlighted different contacts. EEG localizations were mainly clustered near TA contacts, while MEG results were close to the posterior lateral neocortical contacts HA8–10 and, partially, to HP. In agreement with our findings, in temporal lobe epilepsy, posterior MEG source reconstructions in comparison to EEG were also observed in other studies [66], [67]. The main reason for this difference might be the increased size of the active patch at this time point due to propagation. Considering the wide extent of active cortex measured with sEEG in this study, our hypothesis is that the peak of the EEG deviation scan was found at the temporal pole because of its considerable radial source orientation component. As a result, this activation did not contribute much to the MEG signals. On the other hand, the activity arising from especially the posterior lateral neocortical contacts HA8–10 and from the HP contacts was more tangentially oriented, leading to higher SNRs in MEG, and thus MEG was mainly focusing on this part of the cortex. Furthermore, the averaged MEG signal peak was not synchronous with the EEG peak; it preceded the EEG by approximately 7 ms (see dashed vertical line in Fig. 1). In order to determine the sources dominating the EEG and MEG signals, we investigated the time relationship between the peaks of the simultaneously measured sEEG and low-density EEG (ldEEG) epileptic spikes. We observed that the peaks of the ldEEG and the TA4 contact were simultaneous, and our EEG localizations were clustered around TA4. Although MEG and sEEG were not measured simultaneously, we might extrapolate the results of the simultaneous ldEEG-sEEG to the simultaneous EEG-MEG measurements and comment on the timings of the measured signals. Considering the fact that the MEG peak is also approximately 7 ms before the EEG we might state that the MEG maximum is concurrent in time with the HP2 contact. This means, the peaks of the MEG and the HP2 contact are almost simultaneous and might explain why MEG was also localized closer to HP2. All these results fit well to our hypotheses that a larger activated cortical patch is underlying the measured activity and that EEG and MEG focus on only parts and, due to their distinct sensitivities, to non-identical parts of this activity. Although EEG and MEG source reconstructions were able to highlight just a subset of spiking sEEG contacts, EMEG results were covering almost all relevant sEEG contacts with only a few spurious localizations. EMEG localizations were not simply the union of EEG and MEG results but a rather complicated interplay of both modalities compensating their relative shortcomings. For example at -23 ms, in Figs. 6 (especially the lower subfigure) and 7, no major dipole cluster was noticeable neither with EEG nor with MEG around the E contacts in sEEG, while there were clear clusters around these active contacts in EMEG. This also supports the idea that combining EEG and MEG can supply important additional information that cannot be achieved by localizing EEG and MEG alone, and then comparing their results. Therefore, whenever it is technically feasible to measure EEG and MEG simultaneously, it might be important to not only analyze single modality EEG and MEG but also to compare with combined EMEG reconstructions to obtain accurate localization results. Furthermore, the asynchronous EEG and MEG peaks along with the more complete overview on the propagation pathways provided only by EMEG, as shown here, might also help distinguishing between the primary and secondary interictal areas as reported in [68].

Close to the spike peak (-3 ms) the source reconstructions were more anterior in comparison to earlier instants in time and were clustered close to the pole of the temporal lobe. The SNR values shown in the lower subfigure of Fig. 3 support our findings that epileptic activity had started at deeper areas and then propagated to the pole of the temporal lobe at the spike peak. The SNRs of EEG and MEG at spike onset were almost identical, but later on, the increase in EEG SNR was higher than in MEG due to the mainly radial source orientation in the area of the temporal pole.

### Effects of Epileptic Spike Averaging on Source Reconstruction

The decision between localizing each single epileptic spike separately and averaging spikes with similar topographies before source reconstruction is a highly disputed issue in presurgical epilepsy diagnosis, and both approaches have their merits and drawbacks. Single spike localizations might be used to estimate the size of the irritative zone [5], [17], [43–46]. However, these localizations suffer from low SNRs as also shown in our study. On the other hand, averaging similar spikes might increase the SNR and thus, the reliability of the localizations remarkably [2], but information on the extent might get lost. In this paper, motivated by the findings of Bast et al. [2] and Wennberg and Cheyne [69] for EEG, and Wennberg and Cheyne [67] for MEG source reconstructions, we calculated multiple subaverages in order to investigate the effects of SNR and averaging on EEG, MEG and EMEG source reconstructions. This enabled us to compare the effects of averaging and the resulting SNR in a step-by-step fashion.

**Systematic localization bias in single spike source reconstructions due to low SNR**. Our results show that the centroid dipoles obtained from epileptic spike clusters differ considerably between different subaverages. We observed that spikes with lower number of subaverages and thus lower SNRs were localized more mesial and superior in comparison to those with higher number of averages (and higher SNRs) at -23 ms (Fig. 4). The reason for this localization bias might be due to background activity, which can be considered as noise in our case. Since at this time instant the propagation had already occurred, the noise bias shifted the localizations from lateral parts of the temporal lobe into deeper regions in the brain. Our data support this hypothesis by showing higher localization differences in the left-right (LR) and superior-inferior (SI) than in the anterior-posterior (AP) axis. The lateral regions of the left temporal lobe are situated farther away from the center of the brain in LR and SI axes, while in AP axis they are close to the center. In agreement to our results and our hypothesis, the studies of Wennberg and Cheyne [67], [69] also showed similar shifts to the center of the brain.

**Preselection criteria to improve single and subaveraged spike source reconstructions**. Different preselection criteria for epileptic spikes have been suggested to avoid errors in single spike localizations [5], [11], [46]. We followed these criteria and localized only the spikes with SNR higher than three. This preselection strategy resulted in more reasonable localizations but, on the other hand, the number of single spikes that satisfied this condition also got smaller. From the 200 measured single spikes, only 20 EMEG spikes passed the criterion at 0 ms (spike peak with highest SNR). At -23 ms this number was even reduced to only 7 for EMEG and even among them spurious localizations persisted (see EMEG results for Av1 in Fig. 5). Therefore, we recommend (1) to use subaverages and (2) to observe the changes in centroid dipoles and scatter size with increasing averages.

**Estimation of the optimal subaverage number**. For the estimation of the optimal subaverage number, we recommend the following procedure: A subaverage should be selected that averages enough spikes (in our case Av10) so that its centroid dipole does no longer differ much from the centroids of the subaverages with more spikes (in our case Av25 and Av50). Since even for an extended source the center of gravity would always result in the same position in noise free set-up, the changes in the centroid dipole for different subaverages are mainly due to insufficient SNR. By selecting Av10 in which the location of the centroid dipole does not differ much from Av50, we reduced the effects of noise on dipole scatter [70–72]. Averaging more spikes may not be favorable, as this may artificially reduce the scatter size leading to an underestimation of the extent of the irritative zone. Nevertheless, even for the optimal subaverage number estimated with this procedure, the effects of spatial averaging on scatter size will persist and possibly lead to a slight underestimation of the size of the irritative zone. However, the negative influence will be much smaller than localizing single spikes with insufficient SNRs. In this study Av10 was a good compromise, but this number might surely be different for other patients. The better performance of Av10 in comparison to Av50 might be surprising since the higher SNRs of Av50 might be expected to result in better localizations. However, in the light of our results and the relevant literature we can question this expectation at least for localization of interictal spikes: In [73], among 19 patients with Engel I or II outcomes, the resected areas in four cases were concordant to only single spikes, in two to only averaged spikes, and in five to both single and averaged spike localizations. A possible explanation for the latter results can be sought in the light of the publications of [43] and [44], in which using optical imaging they showed that the origins of the epileptic activity change in a stochastic way within a certain region. This questions the assumption that spikes from the same irritative zone have exactly the same origin and waveform, and can be used as an argument against averaging.

**Topology of the irritative zone**. Another important aspect is the topology of the irritative zone. In our study, the irritative zone had a convex shape so that the center of gravity was part of the zone. However, in case of a concave shape, this might change. As an example, the center of gravity of a half-moon-shaped concave topology might be outside the structure (see, e.g., the half-moon-shaped single spike localizations of patient 5 in Fig. 1B in [73]). However, even in the latter case, using the centroid localization change between different subaverages is still an important measure, because a centroid shift between single spike and subaveraged spike localizations will still indicate a systematic shift of single spike localizations due to noise.

**Relationship between size of dipole scatter, SNR and extent of the irritative zone**. Oishi et al. [46] showed in an MEG study that 8 out of 9 patients in which the spike cluster coincided entirely with the ictal onset zone determined by subdural EEG (and resected afterwards) became seizure free whereas the ratio was just 3 out of 11 for the cases where spike cluster and ictal onset zone either only coincided partly or did not coincide at all. In agreement with the studies of [5], [17], [45], [74], this shows the potential benefit of single spike localizations. On the other hand, our results show that the amount of scatter is highly correlated with the number of subaverages and the SNR, especially for relatively low SNRs. Our results on scatter size are mainly in line with Bast et al. [2] and Wennberg and Cheyne [67], [69]. However, we additionally show here that the effects of subaveraging and SNR on scatter size are valid for all investigated modalities, i.e., EEG, MEG and EMEG. Furthermore, while the other studies rely on simpler volume conduction modeling such as spherical shell models used in Bast et al. [2], the high-resolution six-compartment head model with calibrated skull conductivity and anisotropic representation of the white matter compartment as proposed in our study does not only enable simultaneous analysis of EEG and MEG, but also has the potential to improve localization accuracy for single modality EEG or MEG or in combined EMEG analysis. The latter is especially important in the temporal lobe, where a sphere approximation of the skull can result in significant errors for both EEG (e.g., [15]) and MEG (e.g., [75], [76]). Our results are also in agreement with EEG simulations of Kobayashi et al. [77] showing dipole clusters to become less erroneously distributed with increasing SNR. However, in summary, the identification of the exact size of the irritative zone still remains a difficult problem because, as also shown in this study, scatter varies significantly with SNR, spike selection criterion and subaverage number.

Reconstructing slightly distributed activity using a single dipole model might lead to a small depth-bias (sources that are localized slightly too deep). Here, we took three measures to alleviate such depth-bias and to accurately (as validated by the sEEG) reconstruct the center of the underlying activity: 1) We used a cortically-constrained source space, which prevents erroneous localizations inside white matter. 2) We constructed a head model that distinguishes CSF, gray matter, and anisotropic white matter instead of a homogeneous brain, in which the topographies for dipoles with different depths and locations would have been more similar and homogeneous. 3) We preferred a dipole scan instead of a dipole fit to ensure finding the global optimum of the cost function over the cortically-constrained source space.

It is important to state that in this study the aim was not to estimate the extent of a patch in which all neurons are active simultaneously and always in the same way but to estimate the extent of a patch in which the origin of the activity is different for each spike. Using optical imaging on epileptic human neocortical slices removed during epilepsy surgery, Köhling et al. [43] and Speckmann et al. [44] showed that the activated cortical areas during epileptic waves are focal and their spatial positions change in a dynamic manner within the epileptic tissue. This finding was the main reason why a subaveraging procedure was selected instead of averaging all spikes. Therefore, in this study our aim with investigating the dipole scatter was not to determine the extent of a patch that always follows exactly the same activation pattern but to benefit from the small differences on the activation pattern within the epileptogenic zone due to the dynamic and stochastic behavior of each spike as shown in [43,44].

With regard to the chosen inverse approach, besides the cortically-constrained deviation scan as employed here (see, e.g., [38], [55], [56]), promising results were also achieved with current density approaches [38], with hierarchical Bayesian modeling frameworks [48], [78] and with spatio-temporal current density approaches [79], [80] in non-invasively reconstructing networks of (epileptic) activity from EEG and/or MEG. However, also those methods need to embed correct prior knowledge in some form into the inverse approach and it still needs to be shown that the methodology is stable even in the presence of low SNR in realistic epilepsy datasets [78], [79]. Furthermore, in Bouet et al. [81], using frequency domain beamformers, the determination of the spiking volume was possible in 16 out of 21 patients with sensitivity (76%) and specificity (67%), as also validated through sEEG measurements. However, Steinsträter et al. [82] showed that beamformer approaches are sensitive to head volume conductor properties. Therefore, in a future study, it will be interesting to combine other inverse methods with the subaveraging, the head modeling and the combined EEG/MEG procedure as presented here and to evaluate their quality by means of the intracranial EEG recordings.

**Limitations of the current study**. There are two important aspects that need attention regarding the interpretation of the results presented here: 1) We used sEEG measurements for validation purposes because this is widely accepted as the “gold standard” in presurgical epilepsy diagnosis. sEEG has advantages over noninvasive EEG because not only the target sources are closer to the measurement sensors but also the attenuation and smoothing of the signals due to volume conduction, especially due to the highly insulating skull, are avoided. Despite these advantages, sEEG still lacks the ability to show the ground truth due to its low spatial resolution and overall coverage caused by the limited number of electrodes and contacts. 2) Although we have simultaneously measured EEG/MEG and ldEEG/sEEG, we did not measure EEG/MEG/sEEG simultaneously. Thus, we cannot be sure that all epileptic spikes that were visible in sEEG were also visible in EEG and MEG. Nevertheless, we verified that most sEEG spikes were also visible in ldEEG and, as Fig. 7 (EMEG, -23ms) shows, the irritative zones determined by sEEG (green and red contacts) and by noninvasive EEG/MEG (blue dipoles) were well in agreement with each other.

## Conclusions

In this study, a high-resolution realistic six-compartment finite element head model with anisotropic white matter and calibrated skull conductivity enabled us to take into account the different sensitivity profiles of EEG, MEG and EMEG in reconstructing the underlying sources in the brain, and to make reliable interpretations on the effects of spike averaging and SNR. Our study shows that EMEG source analysis can increase accuracy and confidence in source reconstructions significantly, which might have important clinical implications especially for localizing at spike onset and for revealing propagation pathways as complete as possible. Furthermore, subaveraging might provide important and accurate information that neither single nor grand-averaged spike reconstructions can give. However, the extent of dipole scatter will still be correlated to the number of subaverages and to the SNR. Although an advanced head model as used in this study can improve the accuracy of source reconstructions, also studies that use more homogenized ways of forward modeling such as the classical three compartment (skin, skull, brain) approach might still benefit from the subaveraging pipeline and the calibration procedure for combining EEG and MEG as presented in this study.

## Supporting Information

S1 Dataset Dataset underlying the results presented. The spreadsheet contains all deviation scans of EMEG (page 1), EEG (page 2), and MEG (page 3) for subaverages Av5, Av10, Av15, Av20, Av25, Av30, Av35, Av40, Av45, Av50 and single spikes (Av1) at -33, -23, -13, and -3 ms. For each deviation scan signal-to-noise-ratio “SNR”, “Residual variance”, the coordinates of the deviation scan dipole “Locations”, the normals to determine the orientation “Normals”, and the strengths of the dipoles “Strengths” are given. The fourth page contains the names of the sEEG electrodes “Electrode name”, “Contact number”, and their coordinates “Locations”.
(XLSX)

S1 Fig Quantitative comparison of EEG source reconstructions and sEEG contacts at different propagation phases. Square distance indexes and the percentage of dipoles closer than 10 mm for each sEEG contact. The values are given for Av10 EEG subaverages at -33, -23, -13 and -3 ms. The sEEG contacts enclosed by dashed lines were within the seizure onset zone.
(TIF)

S2 Fig Quantitative comparison of MEG source reconstructions and sEEG contacts at different propagation phases. Square distance indexes and the percentage of dipoles closer than 10 mm for each sEEG contact. The values are given for Av10 MEG subaverages at -33, -23, -13 and -3 ms. The sEEG contacts enclosed by dashed lines were within the seizure onset zone.
(TIF)
