# Supplementary figures and images for: Combined EEG/MEG Can Outperform Single Modality EEG or MEG Source Reconstruction in Presurgical Epilepsy Diagnosis
Source: PLoS One. 2015 Mar 11;10(3):e0118753. doi: 10.1371/journal.pone.0118753 (PMC4356563; doi:10.1371/journal.pone.0118753)

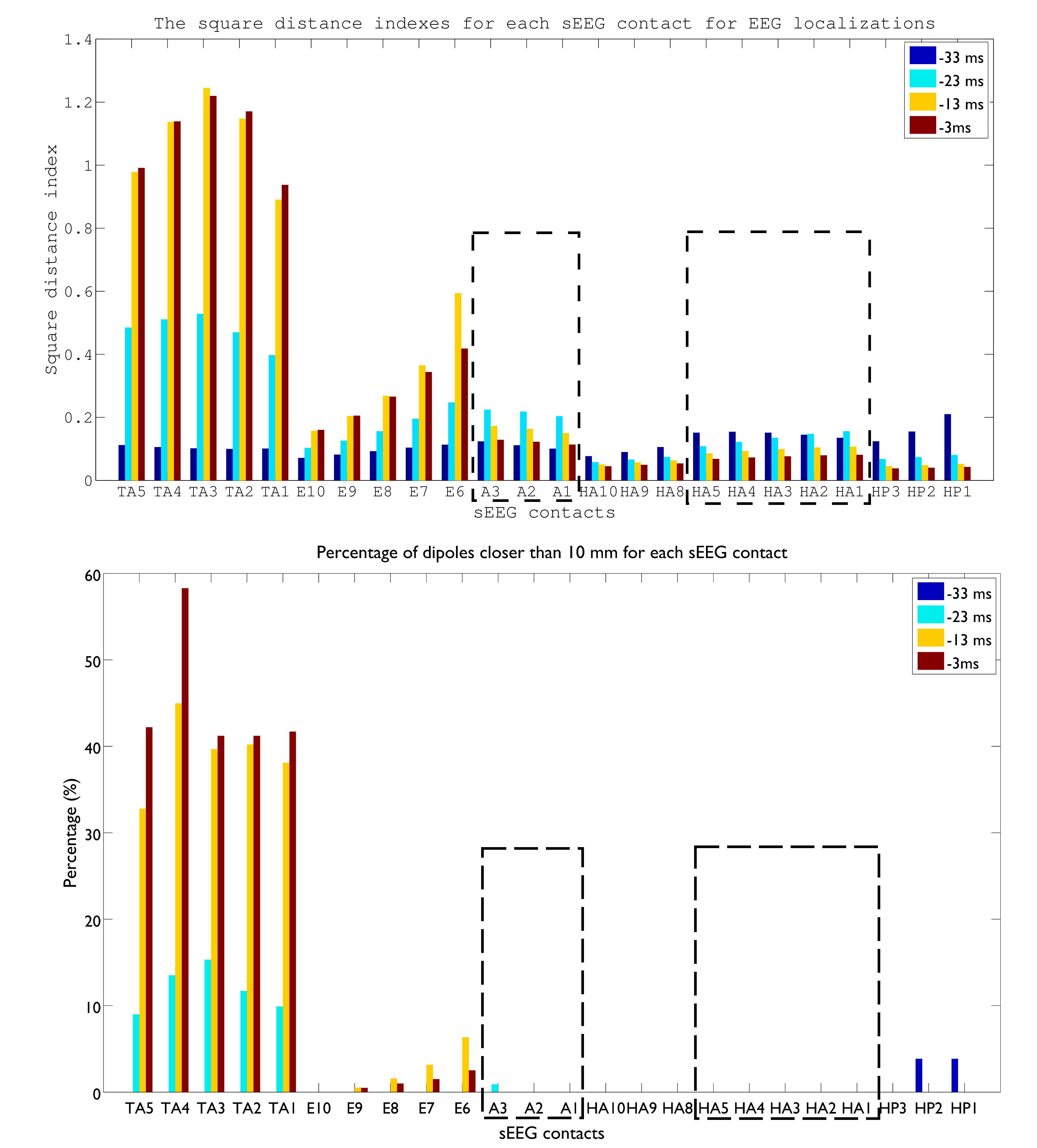

Supplement: S1 Fig — Square distance indexes and the percentage of dipoles closer than 10 mm for each sEEG contact. The values are given for Av10 EEG subaverages at -33, -23, -13 and -3 ms. The sEEG contacts enclosed by dashed lines were within the seizure onset zone. (TIF) [file pone.0118753.s002.tif]

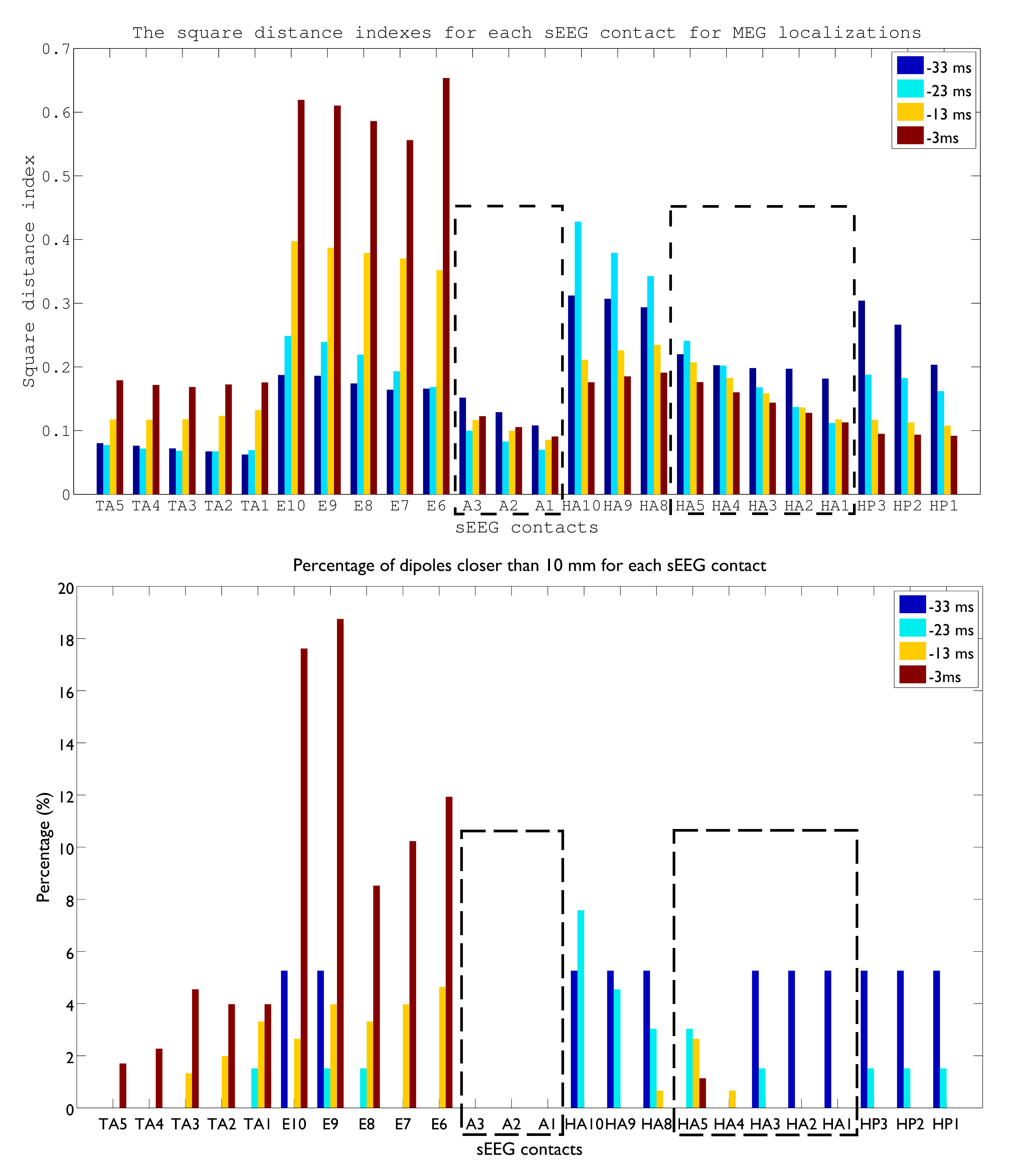

Supplement: S2 Fig — Square distance indexes and the percentage of dipoles closer than 10 mm for each sEEG contact. The values are given for Av10 MEG subaverages at -33, -23, -13 and -3 ms. The sEEG contacts enclosed by dashed lines were within the seizure onset zone. (TIF) [file pone.0118753.s003.tif]
